# Psychometric Properties and Factor Structure of the Malay Autism Spectrum Quotient: Children’s Version

**DOI:** 10.21315/mjms2021.28.6.11

**Published:** 2021-12-22

**Authors:** Shazia Iqbal HASHMI, Getrude Cosmas AH GANG, Agnes SOMBULING, Nurul Hudani MD NAWI, Puteri Hayati MEGAT AHMAD

**Affiliations:** Faculty of Psychology and Education, Universiti Malaysia Sabah, Kota Kinabalu, Sabah, Malaysia

**Keywords:** autism spectrum disorder, screening instrument, psychometric properties, factor structure

## Abstract

**Introduction:**

The Malay autism spectrum quotient, children’s version (AQ-Child) is a translated and reduced version of the autism spectrum disorder (ASD) screening instrument. The aim of this study was to test the reliability and validity of the translated Malay version of the questionnaire.

**Methods:**

The instrument is a 41-item Likert scale form parental questionnaire designed to screen ASD among children aged 6 years old–12 years old. It was filled out by the parents of 700 children (children with ASD = 47; typically developing children = 653) who were contacted through five special education schools and seven mainstream primary schools.

**Results:**

Findings showed that Malay AQ-Child has an internal consistency of 0.82 as a whole scale for 41-items. Receiver operator characteristic analysis showed that the cut score of 63 for the translated, reduced version of the AQ-Child with 41 items had 99% area (95% confidence interval [CI]) under receiver operating characteristic (ROC) curve, and sensitivity and specificity of 93% and 99%, respectively. As for factor structure, principal component analysis (PCA) showed good loading values for most of the items in the instrument.

**Conclusion:**

The translated version of the Malay AQ-Child for screening ASD can further facilitate the process of surveillance and early intervention for children who need it.

## Introduction

The high prevalence rate of autism spectrum disorder (ASD) reported in recent years has created awareness of the importance of screening and surveillance of children at risk for ASD. Therefore, screening for ASD has become an important aspect of early childhood care in most developed countries. For early screening and detection of ASD, developing or translating and validating well-established instruments in different languages has been considered an important responsibility of researchers in different countries. However, studies focusing on available Malay language screening tools for ASD in Malaysia are still limited (1). Thus, this article discusses the findings of the psychometric properties of the Malay autism spectrum quotient, children’s version (AQ-Child), which is the reduced translated version of the autism spectrum quotient (AQ) scale developed for children by Auyeung et al. (2).

The AQ is a screening instrument first developed by Baron-Cohen et al. (3) to study Asperger syndrome and high-functioning autism in adults with normal intelligence. Cross cultural research on ASD using this adult version was conducted with an Australian sample (4), a Dutch sample (5) and a Japanese sample (6), and showed that the scores on the translated versions of this scale were stable among these cultures.

Apart from the adult version of the AQ, an adolescent version of this scale was also developed by Baron-Cohen et al. (3). Both adult and adolescent versions have 50 items that can be grouped together into five distinct categories of autistic traits. These categories are social skills, attention to details, attention switching, communication and imagination. A comparison of the scoring patterns of the AQ adult and adolescent versions revealed similarities between the two groups, and both scales showed good reliability and validity (3).

The AQ-Child version, which is a parent-report questionnaire in the form of a Likert scale (definitely disagree = 3; slightly disagree = 2; slightly agree = 1 and definitely agree = 0) that aims to quantify autistic traits in children 4 years old–11 years old, was developed by Auyeung et al. (2). According to the authors, the AQ-Child has good test/retest reliability and high internal consistency. It has 50 items, so the range of scores on the AQ-Child is from 0 to 150; using the cut score of 76 found high sensitivity and high specificity. However, the items in the AQ-Child version were grouped together into four subscales rather than five subscales like AQ-Adult and AQ-Adolescent. These four subscales are mind reading, attention to detail, social skills and imagination. The translation of AQ-Child into different languages in a recent study by Zhang et al. (7) showed that the Chinese version of AQ-Child is a reliable and valid questionnaire and has good psychometric properties to quantify autistic traits in the mainland Chinese population. Eaves et al. (8) suggest that the Likert response format of AQ-Child is very effective, as it provides parents with several answer choices rather than having only categorical Yes/No response choices that can limit parents’ response options, thus resulting in missing data from skipped items. Apart from that, the Likert response format also helps parents rate the level of difficulty that their children have, which is very useful when screening for ASD to determine the severity of symptoms.

According to the Autism Research Centre website, the AQ-Child version has been translated into 25 different languages, including Arabic, Chinese, Turkish, Spanish, French, Italian and Persian. However, to date, no adaptation and validation of AQ-Child for the Malay-speaking population has been reported; therefore, it was considered important for both ASD screening and research purposes that AQ-Child should be translated and validated in the Malay language. The present research was carried out to determine the psychometric properties of the Malay AQ-Child by assessing its cut-off scores, reliability values and construct validity. Apart from the factor structure, the Malay AQ-Child was also evaluated using principle component analysis (PCA). We assumed that the Malay AQ-Child validated with local samples can be used as a screening tool for ASD in play schools, kindergartens, primary schools, and other pre-school and early childhood care facilities. An early screening process can facilitate and help provide early intervention programmes, which can reduce the effects of the disorder, along with overcoming the factors related to the development of secondary disability.

## Methods

### Research Design

A survey research design was used and AQ-Child was administered as a survey questionnaire. The parents of children from selected schools were contacted and given AQ-Child along with informed consent forms. In this case, implied consent to participate in the study was obtained when they completed and returned the questionnaire (9).

### Instruments

The AQ-Child is a 50-item parent-report questionnaire in the form of a Likert scale (definitely disagree-definitely agree) for measuring autistic traits in children aged 4 years old–11 years old. These items can be grouped together into five difficulty areas associated with ASD: communication, attention to details, social skills, imagination and attention-switching. The scale also has reverse items (1, 3, 8, 10, 11, 14) and considerations were taken while scoring these items. Overall, the score range is from 0–150, with higher scores (76 and above) considered as having ASD. To retain the originality of the actual constructs, AQ-Child was translated to the Malay language using the back-to-back translation method and was named the Malay AQ-Child. Malay AQ-Child was first administered to a small group of participants (n = 35), and parents with children between the ages of 6 years old and 12 years old were contacted from one primary school around Kota Kinabalu for a pilot study. The aim was to test its suitability with Malaysian culture as well as to determine the comprehension and understanding level of parents pertaining to AQ-Child. Further modifications to the Malay version were carried out after analysing the data collected during the pilot study and the comments provided by the respondents. The Malay AQ-Child, along with the biodata form, which requires information such as a child’s age, gender, race, birth order, twin or single birth, was then used in the actual study to collect data.

### Participants and Location

Altogether, parents of 700 children (children diagnosed as having ASD = 47; typically developing children = 653) between the ages of 6 years old–12 years old took part in this study and completed the research questionnaires about their children along with the informed consent forms. In the current study, the sampling frame was acquired from primary schools in the Kota Kinabalu city area. The sample was chosen using a purposive design that involved mainstream primary schools that also provided special education classes and had experienced special education teachers for ASD children. The parents were contacted by obtaining permission from the teacher through five special education classes and seven mainstream primary school classes around Kota Kinabalu, a Malaysian city area.

### Instrument Translation and Cross-Cultural Adaptation

The AQ was translated into Malay using the back-to-back translation method to retain the originality of the actual constructs of the scales. The first step was to translate the original English questionnaire to Malay using two bilingual translators of local Sabahan descent. The second step in the translation process involved combining these questionnaires. After the Malay versions of the questionnaires were ready, the process of further reviewing and combining was carried out by the team of Malay academician who was bilingual. This step was taken to ensure that the combined version was consistent with the original version. Important notes about appropriate wordings and terms were taken during this stage. The third step was to back-translate the questionnaire. Two bilingual translators who were fluent in both languages back-translated the Malay version of the questionnaire into English. Neither of the translators was given any original version of the questionnaire and they worked independently. The English versions were reviewed again to determine the accuracy of the translation process. Important notes about appropriate wording and terms were taken during this stage as well. On the basis of differences or similarities between the original English version and the back-translated English versions, conclusions were drawn about the equivalence of both versions. Minor changes were made to the Malay questionnaire after the back-translation process. The finalised Malay version of the questionnaires was further edited to minimise spelling and grammatical errors.

### Data Analysis

The data were analysed using SPSS 24. Descriptive statistics were used to analyse demographic information. Receiver operating characteristic (ROC) curve analyses were conducted to examine the area under the ROC curve. Sensitivity and specificity values to determine the range of potential cut-off scores were also calculated. The reliability of the scales was determined by Cronbach’s alpha and PCA was conducted to determine the factor structure of the items in the scale.

### Procedure

Upon seeking ethical approval from relevant authorities, a discussion was held between the research team and the selected schools’ administration to explain the objectives and flow of the research. After submitting an official letter of application to school administration and receiving formal approval from each school, further discussions were held among the research team and class teachers regarding the flow of research. Respective class teachers were requested for their cooperation and support during the data collection phase. Research questionnaires, along with informed consent forms, were distributed with the help of class teachers on the first day of the week by research team members to the parents of children who had established diagnoses of ASD by medical practitioners and were identified by their class teachers. All these children were holders of the person with disabilities card (*kad orang kurang upaya*) issued by the Malaysian Social Welfare Department. The questionnaires were collected after three days. Based on Kriejcie and Morgan (10), 63 questionnaires were distributed to parents of children from special education classrooms, 54 (85%) were collected back and 47 (74.6%) were analysed. The same procedure was followed to collect data from parents of typically developing children. Overall, 770 questionnaires were distributed among children from mainstream classes; about 674 (87.5%) were returned, which was quite satisfactory. However, after removing incomplete questionnaires, only 653 (85%) were analysed. The completion ratio was 14:1, and the results were analysed for typically developing children and children with ASD. According to the Diagnostic and Statistical Manual of Mental Disorders, 5th Edition (DSM-5), the prevalence of ASD has been reported to be approximately 1% of the population; therefore, the representation of typically developing children and children with ASD seems adequate for this research.

### Establishing Validity

Exploratory factor analysis (EFA) is a statistical data reduction technique used to explain variability among observed constructs, which is part of the factor analysis technique introduced by Thurstone (11). The analysis seeks to uncover the underlying factors that explain the dimensionality of each construct in the research. The analysis assumes that all rating data on different attributes can be reduced to a few important dimensions or components. The reduction is possible because the attributes are related. This statistical technique will deconstruct the raw score into various components and reconstruct the score into the underlying factor score. The degree of correlation between the initial raw score and the final factor score is known as factor loading.

EFA was conducted using SPSS to examine the factor structures as a preliminary step in understanding the clustering of the items. In other words, it was used to determine whether items that together measure a construct load highly with the same factor. Only valid items were used for subsequent analysis. The EFA procedure uses the PCA extraction method with Varimax rotation. However, to determine whether the data were suitable for factor analysis, satisfaction of the following conditions to measure sampling adequacy was considered: i) Kaiser-Meyer Olkin (KMO) test’s score > 0.6 and ii) Bartlett’s test of sphericity, significant value: *P* < 0.05. In this case, the Bartlett’s test of sphericity was significant (KMO test score > 0.6) and reflected that the current data was adequate to proceed to EFA.

The next course of action was to examine the factor loadings of each item. If all items converged into the same factor, we assumed that the factor was to be constructed. According to Hair et al. (12), the retention criteria was indicated by an item of factor loading ≥ 0.5. If it was lower than this standard value or the item cross-loaded into other factors, then it was dropped from further analysis.

### Establishing the Reliability of the Scale

Reliability is another important aspect of accessing the quality of measurement instruments. Reliability refers to an estimation of the degree to which a measurement is free from errors. According to Sekaran (13), the reliability of a measure indicates the extent to which it is without bias (error free), and hence ensures consistent measurement across time and across various items in the instruments. In other words, it is an indication of the consistency and stability with which the instrument measures the concept and helps to assess the ‘goodness’ of a measure. Stability of measures can be achieved from test-retest reliability and parallel form reliability. In this context, the same measure is considered stable if the scores are obtained from the same set of respondents at different times or from different sets of forms with changes in terms of the wording and sequence of the question. However, the consistency of the measure can be determined from inter-item consistency reliability and split-half reliability.

Therefore, the reliability test was conducted in this research to ensure that there was an acceptable internal consistency among the items that represented a particular factor, and Cronbach’s alpha, one of the most commonly used reliability coefficients, was applied.

## Results

### Biodata of Children Reported by Parents

Table 1 shows the biodata profiles of children reported by their parents. Although there were more female students than male students in mainstream schools, gender differences in special education classes had males outnumbering females, with a ratio of 1:5.5. Mainstream school ages ranged from 6 years old–10 years old, while in special education classes, ages ranged from 7 years old–12 years old. Both mainstream schools and special education classes had a high representation of Bumiputra Sabah*.*

### Exploratory Factor Analysis Procedure for the Malay Autism Spectrum Quotient, Children’s Version

The EFA was conducted using SPSS version 24 by passing through several stages. Therefore, the Malay AQ-Child construct has 50 measuring items renamed as AQ1 until AQ50 (Table 2). Every item was measured using an interval scale between 0 and 3 (definitely agree = 0; slightly agree = 1; slightly disagree = 2 and definitely disagree = 3) with the item statement for measuring autistic traits in children aged 4 years old–12 years old. The mean score for every item on the Malay AQ-Child is presented in Table 2.

However, item analysis conducted to examine scoring patterns on individual items found that there were nine items (items 9, 13, 21, 23, 24, 29, 30, 43 and 49) where the mean score of typically developing children (TD) was higher than that of children with ASD, which indicated that these items may be difficult to examine and differentiate between young typically developing children and children with ASD. Therefore, in line with the findings of Auyeung et al. (2), the decision was made by the research team to eliminate these items while carrying out the factor analysis.

The EFA procedure using the PCA extraction method with Varimax rotation was carried out on 41 items measuring AQ-Child construct. Table 3 shows the value for Bartlett’s test of sphericity, which was significant (*P* < 0.05), and the measure of sampling adequacy by KMO test was 0.823, which was higher than the minimum requirement of 0.6 (13). Both values (Bartlett’s test of sphericity, which was significant and KMO test > 0.6) reflected that the current data was adequate to proceed into the next step, the EFA (13).

### Factor Structures of the Malay Autism Spectrum Quotient, Children’s Version

The 41 items of the Malay AQ-Child were subjected to PCA, which was conducted to determine the factor structure of the items in the scale. The suitability of AQ-Child data for factor analysis revealed that regarding measuring of sampling adequacy, the KMO test was 0.82 and the Bartlett’s test of sphericity achieved statistical significance (χ^2^ = 7104.683, *df* = 820*, P* < 0.001). The result, as shown in Table 3, indicated that the data was still suitable for factor analysis, since the value of KMO test was greater than 0.6, and Bartlett’s test of sphericity was significant (*P* < 0.000).

**Figure 1:**
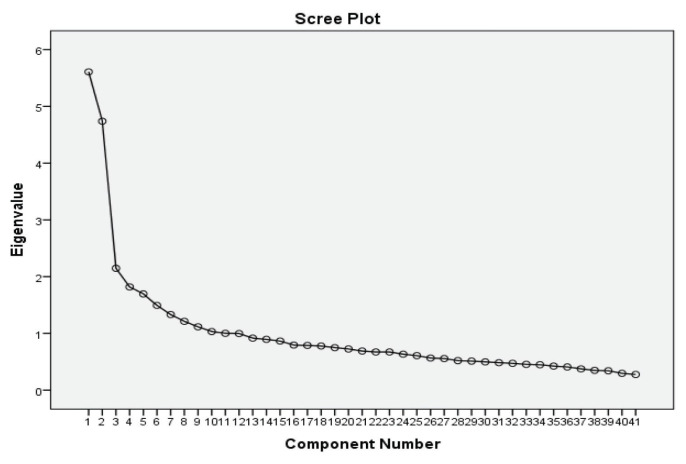
Scree plot with break after first and second component

Based on the two main categories of symptoms of ASD, as suggested in DSM-5 and the scree plot, presented in Figure 1, only two components were retained. The outputs based on Table 4 show that component 1 contributed 10.128% and component 2 contributed 9.113%, respectively. The total variance explaining the construct was acceptable, since it exceeded the minimum requirement of 60% (12). Values lower than 60% would indicate that the existing items were not adequate to measure the construct.

Applying the two-component factor structure fit used by Vulchanova et al. (15), the two components retained during the current result analyses showed moderate to good loading for the 37 items, which ranged from 0.615 to 0.316 for component 1 and 0.673 to 0.313 for component 2. Further examination revealed that items loaded on component one were more related to social communication and social interactions, while items on component two were mostly related to restricted, repetitive patterns of behaviour; thus, the components were named accordingly. Apart from that, the component matrix table showed that items 25, 31, 37 and 41 were not loaded into any of the two retained components. Table 5 presents the factor structure for the Malay AQ-Child.

### Cut-off Scores for the Malay Autism Spectrum Quotient, Children’s Version

**Figure 2:**
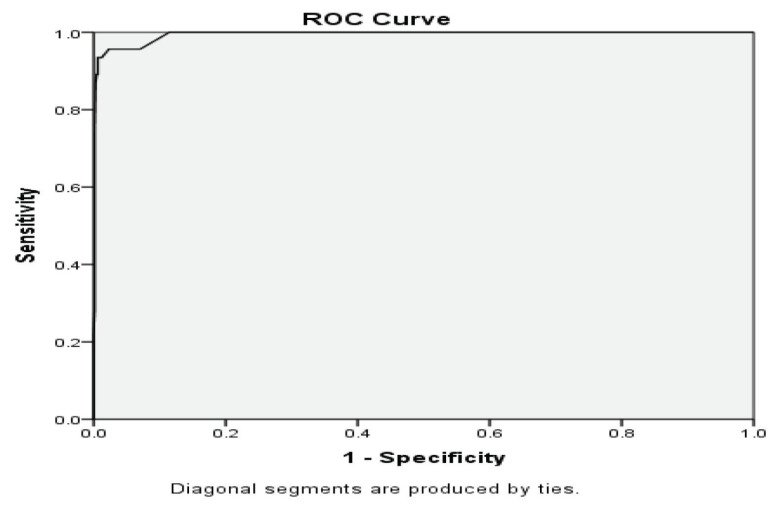
ROC curve of the sensitivity and specificity of the Malay AQ-Child score. Area under the curve = 0.94

The area under the ROC curve for the Malay AQ-Child was 0.99 (95% CI), which shows that the score on this instrument is a good indicator of ASD. Figure 2 shows that the ROC analysis further suggested that the cut-off score of 63 showed high sensitivity (93%) and high specificity (99%). Table 6 presents a comparison of cut-off points for the Malay AQ-Child.

**Figure 3:**
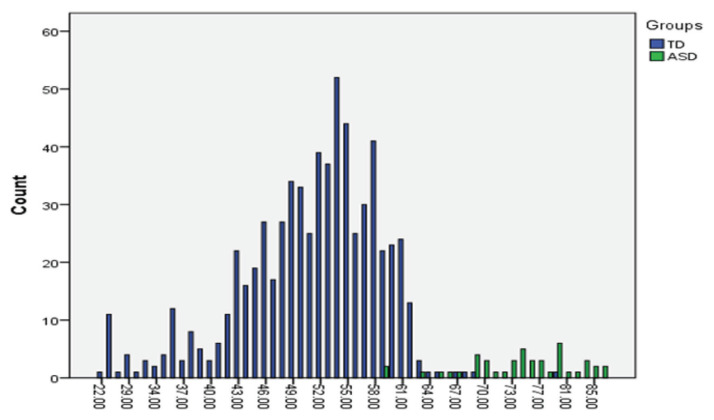
Scoring pattern on AQ-Child by groups

Furthermore, the scoring pattern displayed on the AQ-Child by both groups, as presented in Figure 3, showed that 95% of children with ASD scored at or above the suggested cut-off point of 63, whereas for typically developing children, only 1.5% did so, and the rest (98.5%) scored below this value. These results also suggest that the Malay AQ-Child has adequate construct validity, as participating children with established ASD diagnoses scored higher on the scale compared with typically developing children.

### Reliability of Malay Autism Spectrum Quotient

The reliability test is conducted to ensure that there is an acceptable internal consistency among the items that represent a particular factor and Cronbach’s alpha is one of the most commonly used as reliability coefficients. The results presented in Table 7 showed that the Malay AQ-Child had good internal consistency values based on the Cronbach’s alpha for the whole scale and for items arranged in two different subscales, according to the difficulty areas associated with ASD. It shows that Cronbach’s alpha score exceeded the lower limit on reliability of 0.70. This indicates a high level of internal consistency among the instruments.

## Discussion

The aim of this research was to examine the psychometric properties and factor structure of the Malay AQ-Child’s version. The AQ has 50 items in a Likert scale format developed to screen for ASD among adults (AQ-Adult) (3), adolescents (AQ-Adolescent) (14) and children (AQ-Child) (2). According to these researchers, score significantly higher than the suggested cut-off point is associated with ASD. However, based on the examination of the mean score for each item obtained during the current research, typically developing children scored higher on nine items (9, 13, 21, 23, 24, 29, 30, 43 and 49) compared to children with ASD; thus, these items were not included in further analysis. Careful examination revealed that these items were related to memory, preference for reading books (fiction), planning activities, and preferred places to go, including parties, libraries, theatres and museums, which are activities that may not be suitable for young children. Auyeung et al. (2) found that children from the control group scored higher on items 29, 30 and 49, and thus removed these items from subsequent analyses.

A cut-off score of 63 with 41 retained items was found to have high sensitivity (93%) and high specificity (99%) values, and the Malay AQ-Child had good construct validity, as those with established ASD diagnoses scored significantly higher than those without the diagnosis. However, researchers and clinicians must be careful when using the cut-off points during the screening phase, as showing a trait does not necessarily indicate a tendency to have a disorder, and apart from including other relevant criteria, the presence of a disorder should also be accompanied by impairment in everyday functioning.

The two-component factor structure of the Malay AQ-Child showed that most of the item loadings were moderate to good. The two components factor structure also had the best data fit, as stated by Vulchanova et al. (15) while exploring the factor structure of the Bulgarian Childhood Autism Spectrum test, which is a 37-item parental questionnaire developed by Scott et al. (16) for screening ASD among children in the UK. They further suggested that this two-component structure is consistent with the two main symptom categories adopted in the DSM-5 as diagnostic criteria for ASD.

The present research also found that the Malay AQ-Child had adequate reliability for the whole scale and the two subscales based on components retained during factor analysis. These results are consistent with the findings by Zhang et al. (7). By determining the psychometric properties of the Chinese AQ-Child for mainland China in both clinical and non-clinical samples, the authors also found that the Chinese AQ-Child has good to excellent reliability values for the whole scale and subscales. According to Auyeung et al. (2), AQ-Child has high coefficient values for the scale and subscales and it has excellent test/retest reliability. Wakabayashi et al. (17) also reported good to excellent reliability values for the Japanese AQ-Child. Thus, based on the findings of the present research and the work of other researchers on AQ-Child, this instrument will provide reliable scores in the future if used for screening ASD among children aged 6 years old-12 years old in a large population-based study in Malaysia. Therefore, this study concludes that the instrument chosen during the present research was a reliable and valid measure after translation into Malay and can be used in the Malaysian context.

The findings of the present research indicate that Malay AQ-Child can be used in the local Malaysian context, in which there is a need for screening tools for autism in the local language. However, caution must be taken as adapting screening instruments developed for another culture can pose some challenges. As every culture has its unique way of understanding and explaining human behaviour, some of the behaviours that might be considered appropriate in one culture may not be considered the same way in other culture. For example, non-verbal communication bears different meanings in different cultures. In the context of the current research, ‘lack of eye contact’, which is presented as one of the defining clinical features of ASD, is viewed differently among Asian cultures, as looking directly into someone’s eyes is not considered socially appropriate behaviour in many Asian cultures. Further, future potential users of Malay AQ-Child must be cautious; although this questionnaire might be useful in screening ASD, it cannot serve as an alternative for a clinical diagnosis.

## Conclusion

The Malay AQ-Child, validated with local samples in the present research, can be used as a meaningful screening instrument for ASD in various early childhood care facilities, including kindergartens, playschools and primary schools. The findings of the present research highlight the Malay AQ-Child as an instrument that is reliable and valid; therefore, its use in clinical and research settings will help professionals screen for the presence of ASD. Future research should further evaluate how the Malay AQ-Child performs on a national scale in studies that screen large populations. As the main objective of the study was to translate the scales and determine their basic psychometric properties, future cross-linguistic and cross-cultural research could explore other aspects of these translated versions of the instruments to reveal the extent to which our findings can be applied and used in other contexts.

## Figures and Tables

**Table 1:** Biodata profile information of research participants from mainstream schools and special education

Variables	Mainstream schools (*n* = 653)		Special education (*n* = 47)	

	Frequency	Percentage	Frequency	Percentage
Gender				
Male	317	48.5	40	85
Female	336	51.5	7	15
Age (years old)				
6	2	0.3	0	0
7	238	36.4	9	19.2
8	210	32.2	10	21.27
9	202	31.0	10	21.27
10	1	0.2	9	19.2
11	0	0	4	8.5
12	0	0	5	10.5
Ethnicity				
Malay	164	25.2	3	6.38
Chinese	4	0.6	2	4.25
India	2	0.3	0	0
Bumiputra Sabah	431	66.0	35	74.46
Bumiputra Sarawak	12	1.9	2	4.25
Others	39	6.0	5	10.5

**Table 2:** Mean scores obtained for each item

Items	TD children	ASD children
AQ1 Prefer do things with others rather than on her/his own.	1.22	1.87
AQ2 Do things the same way over and over.	1.43	2.45
AQ3 Finds it very easy to create a picture in her/his mind	0.87	1.17
AQ4 Strongly absorbed in one thing that loses sight of other things	1.49	2.28
AQ5 Notices small sounds when others do not	1.23	2.09
AQ6 Notices numbers or strings of information	1.66	1.83
AQ7 Is impolite, even though s/he thinks it is polite	0.93	2.02
AQ8 Can easily imagine what story characters look like	0.85	1.49
AQ9 Fascinated by dates	1.68	1.34
AQ10 Can easily keep track of several conversations	1.05	2.06
AQ11 Finds social situations easy	1.12	1.76
AQ12 Notices details that others do not	1.73	1.93
AQ13 Would rather go to a library than a party	1.36	1.00
AQ14 Finds making up stories easy	1.38	1.74
AQ15 Drawn more strongly to people than to things	1.57	1.97
AQ16 Tends to have very strong interests	1.86	2.34
AQ17 Enjoys social chit-chat	1.00	1.85
AQ18 Does not let others to get a word in edgeways	0.88	2.21
AQ19 Fascinated by numbers	1.51	1.74
AQ20 Finds it difficult to work out the characters’ intentions	1.12	2.00
AQ21 Does not particularly enjoy reading fiction	1.55	1.42
AQ22 Finds it hard to make new friends	0.91	1.72
AQ23 Notices patterns	1.88	1.87
AQ24 Would rather go to the theatre than the library	1.81	1.80
AQ25 Gets upset when daily routine is disturbed	1.59	1.78
AQ26 Does not know how to keep up a conversation	0.89	2.04
AQ27 Finds it easy to ‘read between the lines’	1.46	2.14
AQ28 Concentrates on the whole picture rather than details	1.17	1.29
AQ29 Not very good at remembering phone numbers	1.63	1.27
AQ30 Does not usually notice small changes	1.70	1.46
AQ31 Knows how to tell if someone bored	1.23	1.78
AQ32 Finds it easy to do more than one thing at once	1.07	1.31
AQ33 Does not know when it is their turn on the phone	1.13	1.80
AQ34 Enjoys doing things spontaneously	0.87	0.95
AQ35 Often the last to understand a joke	0.87	1.55
AQ36 Finds it easy to work out feelings by looking at faces	1.21	1.65
AQ37 Can switch back after an interruption	1.15	1.29
AQ38 Good at social chit-chat	1.13	2.06
AQ39 Keeps going on and on about the same thing	0.96	1.87
AQ40 Enjoyed playing games involving pretending	0.94	1.89
AQ41 Likes to collect information	1.81	1.87
AQ42 Finds it difficult to imagine being someone else	1.34	1.63
AQ43 Likes to plan activities carefully	1.86	1.42
AQ44 Enjoys social occasions	0.98	1.46
AQ45 Finds it difficult to work out people’s intentions	1.60	1.72
AQ46 New situations make him/her anxious	1.36	1.91
AQ47 Enjoys meeting new people	0.88	1.57
AQ48 Is a good diplomat	1.06	1.93
AQ49 Not very good at remembering people’s date of birth	1.65	1.23
AQ50 Finds it easy to play games that involve pretending	0.87	1.74

**Table 3:** KMO test and Bartlett’s test of sphericity for AQ-Child

Dimension	KMO test	Bartlett’s test	Suitability
AQ-Child	0.823	0.000	Suitable

**Table 4:** Total variance explained contribution by every component

Component	Initial Eigenvalues			Rotation sums of squared loadings		

	Total	% of variance	Cumulative %	Total	% of variance	Cumulative %
1	1.519	10.128	50.746	1.519	10.128	50.746
2	1.367	9.113	59.858	1.367	9.113	69.858
3	0.973	6.487	66.345			

**Table 5:** Factor structure of the Malay AQ-Child

Item	Content	Loading
Social communication and social interactions, Eigenvalue = 5.61, % variance = 13.68		
AQ1	Prefer do things with others rather than on her/his own *(Adakah anak anda lebih suka melakukan sesuatu perkara dengan orang lain berbanding dengan diri sendiri)*	0.414
AQ3	Finds it very easy to create a picture in her/his mind *(Sekiranya anak anda cuba membayangkan sesuatu, dia dengan mudahnya dapat mencipta gambaran dalam mindanya)*	0.519
AQ5	Notices small sounds when others do not *(Anak anda sering mengenalpasti bunyi-bunyi kecil yang tidak dapat dikesan oleh orang lain)*	−0.378
AQ6	Notices numbers or strings of information	−0.437
AQ8	Can easily imagine what story characters look like	0.527
AQ10	Can easily keep track of several conversation	0.615
AQ11	Finds social situations easy	0.558
AQ12	Notices details that others do not	−0.472
AQ14	Finds making up stories easy	0.385
AQ16	Tends to have very strong interests	−0.326
AQ17	Enjoys social chit-chat	0.578
AQ19	Fascinated by numbers	−0.348
AQ27	Drawn more strongly to people than to things	0.415
AQ28	Concentrates on the whole picture rather than details	0.316
AQ32	Finds it easy to do more than one thing at once	0.456
AQ34	Enjoys doing things spontaneously	0.577
AQ36	Finds it easy to work out feelings by looking at faces	0.443
AQ38	Good at social chit-chat	0.537
AQ40	Enjoyed playing games involving pretending	0.448
AQ44	Enjoys social occasions	0.526
AQ47	Enjoys meeting new people	0.543
AQ48	Is a good diplomat	0.344
AQ50	Finds it easy to play games that involve pretending	0.428
**Item**	**Content**	**Loading**
Restricted, repetitive pattern of behaviour, Eigenvalue = 4.74, % variance = 11.55		
AQ2	Do things the same way over and over again.	0.573
AQ4	Strongly absorbed in one thing that loses sight of other things	0.657
AQ7	Is impolite, even though s/he thinks it is polite	0.657
AQ15	Drawn more strongly to people than to things	−0.313
AQ18	Does not let others to get a word in edgeways a story	0.621
AQ20	Finds it difficult to work out the characters’ intentions in a story	0.599
AQ22	Does not know how to keep up a conversation	0.422
AQ26	Does not know how to keep up a conversation	0.600
AQ33	Does not know when it is their turn on the phone	0.500
AQ35	Often the last to understand a joke	0.673
AQ39	Keeps going on and on about the same thing	0.643
AQ42	Finds it difficult to imagine being someone else	0.373
AQ45	Finds it difficult to work out people’s intentions	0.440
AQ46	New situations make him/her anxious	0.467

**Table 6:** Cut-off points for Malay AQ-Child

Indices	Malay AQ-Child cut-off point		

	53	63	73
Sensitivity	1	0.93	0.47
Specificity	0.69	0.99	0.99

**Table 7:** Reliability of Malay AQ-Child

Scale	Items	Cronbach’s alpha
Malay AQ-Child	41	0.81
Social communication and social interactions	23	0.73
Restricted, repetitive pattern of behaviour	14	0.82
